# A pH-Responsive Polycaprolactone–Copper Peroxide Composite Coating Fabricated via Suspension Flame Spraying for Antimicrobial Applications

**DOI:** 10.3390/ma17112666

**Published:** 2024-06-01

**Authors:** Tingting Cui, Daofeng Zhou, Yu Zhang, Decong Kong, Zhijuan Wang, Zhuoyue Han, Meiqi Song, Xierzhati Aimaier, Yanxin Dan, Botao Zhang, Hua Li

**Affiliations:** 1 Cixi Biomedical Research Institute, Wenzhou Medical University, Wenzhou 325035, China; cuitingting@nimte.ac.cn (T.C.); zhoudaofeng@nimte.ac.cn (D.Z.); zhangyuygs@nimte.ac.cn (Y.Z.); kongdecong@nimte.ac.cn (D.K.); wangzhijuan@nimte.ac.cn (Z.W.); hanzhuoyue@nimte.ac.cn (Z.H.); songmeiqi@nimte.ac.cn (M.S.); xierzhati@nimte.ac.cn (X.A.); 2 Institute of Biomedical Engineering, Ningbo Institute of Materials Technology and Engineering, Chinese Academy of Sciences, Ningbo 315201, China; 3 Graduate School of Engineering, Tohoku University, Sendai 980-8577, Japan; dan.yanxin.c2@tohoku.ac.jp; 4 Zhejiang-Japan Joint Laboratory for Antibacterial and Antifouling Technology, Ningbo Cixi Institute of Biomedical Engineering, Ningbo 315201, China

**Keywords:** polycaprolactone, copper peroxide, pH-responsive, antibacterial, coating

## Abstract

In this study, a pH-responsive polycaprolactone (PCL)–copper peroxide (CuO_2_) composite antibacterial coating was developed by suspension flame spraying. The successful synthesis of CuO_2_ nanoparticles and fabrication of the PCL-CuO_2_ composite coatings were confirmed by microstructural and chemical analysis. The composite coatings were structurally homogeneous, with the chemical properties of PCL well maintained. The acidic environment was found to effectively accelerate the dissociation of CuO_2_, allowing the simultaneous release of Cu^2+^ and H_2_O_2_. Antimicrobial tests clearly revealed the enhanced antibacterial properties of the PCL-CuO_2_ composite coating against both *Escherichia coli* and *Staphylococcus aureus* under acidic conditions, with a bactericidal effect of over 99.99%. This study presents a promising approach for constructing pH-responsive antimicrobial coatings for biomedical applications.

## 1. Introduction

In recent years, bacterial resistance infections have emerged as a significant global health challenge. Biomaterial-associated infections pose a serious threat to global human health [1]. Bacterial resistance to antibiotics can be acquired through mutations in the chromosomal genes or the horizontal transfer of resistance genes, resulting in infections that are difficult to treat [2]. In the medical field, the development of chronic wounds occurs when the healing process of hemostasis, inflammation, hyperplasia, and re-epithelialization is not completed promptly following a skin injury [3,4]. Bacterial infection poses a significant challenge in the management of chronic, non-healing wounds [5]. The interplay between the extended healing time of a wound, which heightens the risk of bacterial infection, and the presence of bacterial infection, particularly drug-resistant strains, which in turn delays wound healing, constitutes a challenging aspect of the management of chronic, hard-to-heal wounds. Therefore, there is a long-standing need for the development of innovative antibacterial materials [6]. These materials may include surface coatings, nanoparticles, or hydrogels designed to overcome the resistance of these microorganisms or enhance the efficacy of antibiotic therapy when used in combination. Metal nanoparticles are currently under investigation for their antimicrobial properties and have shown promise as effective antibacterial agents. Balcucho et al. utilized copper oxide (CuO) metal nanoparticles to fabricate composites capable of releasing Cu^2+^ ions, which showed remarkable growth inhibition of methicillin-resistant *Staphylococcus aureus*, exceeding four logarithms [7].

Among the various strategies explored, the catalytic treatment of metal peroxide nanoparticles based on the in situ Fenton reaction has garnered considerable attention as a promising antibacterial approach [8,9,10]. The mechanism underlying the Fenton reaction involves the conversion of hydrogen peroxide (H_2_O_2_) to highly reactive hydroxyl radicals (•OH). It can result in oxidation damage to the membrane and the cell wall and display high and broad-spectrum antibacterial activity compared with traditional antibiotics [11]. Several metal peroxide nanoparticles, such as zinc peroxide (ZnO_2_) and calcium peroxide (CaO_2_), have been constructed as Fenton reaction–based chemodynamic therapy (CDT) agents [12,13]. Recently, Lin et al. first reported the successful synthesis of CuO_2_ nanodots, which could self-supply H_2_O_2_ in the acidic environment and produce highly toxic •OH via the Fenton reaction between Cu^2+^ and H_2_O_2_ [14]. CuO_2_ is a copper oxide with a unique structure that contains Cu^2⁺^ and O_2_^2−^ ions in its molecular structure. It has a bent, end-on structure with inequivalent oxygens and peroxide-like O—O distances, typically 1.4–1.55 Å. It maintains the same spin multiplicity as Cu and CuO and presents a controversial ground state in the neutral 3d-metal dioxide series [15]. CuO_2_ is synthesized from H_2_O_2_ and Cu^2+^ under alkaline conditions. Under weak acid conditions, CuO_2_ could reversibly decompose into Cu^2+^ and H_2_O_2_, leading to a Fenton-like reaction between these decomposition products, which in turn generates reactive oxygen species [14]. Both Cu^2+^ and H_2_O_2_ are well-known antimicrobial agents and have been extensively studied for bacterial infection control. Unlike antibiotics, Cu^2+^ and H_2_O_2_ are not susceptible to bacterial resistance [16]. CuO_2_ demonstrated strong antibacterial effects for biofilm treatment and wound healing [17,18]. The initial environment at the site of bacterial infection is weakly acidic [19], which favors the dissociation of CuO_2_ and enables the application of the pH-responsiveness of CuO_2_.

However, the inherent instability of CuO_2_ under neutral conditions significantly limits its practical application [20]. Additionally, the indiscriminate nature of hydroxyl radicals produced via the Fenton reaction may pose risks of off-target cytotoxicity and tissue damage, highlighting the need for targeted delivery and controlled release strategies to minimize adverse effects. Studies have shown that encapsulation can significantly improve the stability of CuO_2_ [18,20]. Compared to other drug delivery systems (such as nanoparticles, electrostatic spinning [21,22], hydrogels [23], gelatin sponges [24,25], etc.), coatings have higher drug-carrying abilities, are easy to store, and can be used to treat large bacterial infections. The thermal spray processes for polymer coating production include flame spraying, high-velocity oxygen fuel (HVOF)/high-velocity air fuel (HVAF), plasma spraying, and cold spraying [26]. Coating biodegradable polymers by flame spraying is a widely used method, with advantages including low cost, simplicity, and environmental friendliness [27].

Polycaprolactone (PCL) is a hydrophobic polyester that has garnered considerable attention in various biomedical applications. This is primarily due to its exceptional biocompatibility, ability to blend with other polymers, distinctive rheological properties, and controlled release of active compounds. These characteristics are closely tied to its biodegradability [7,28]. Therefore, PCL presents itself as a promising choice for integration as a structural component of dressings that come into direct contact with living tissue. The incorporation of CuO_2_ nanoparticles into a PCL matrix not only preserves the intrinsic properties of the nanoparticles but also extends their stability and facilitates their controlled release [29].

This study focuses on developing a biodegradable and biocompatible material for antimicrobial applications in the weakly acidic microenvironment. An innovative approach using the suspension flame spraying method was employed to fabricate pH-responsive antimicrobial coatings with different contents of CuO_2_ nanoparticles in the PCL matrix. Various analyses were conducted to confirm the successful incorporation of CuO_2_. Their release mechanisms and antimicrobial properties were also examined under different pH conditions. The study offers a universal fabrication method for pH-responsive CuO_2_-loaded composite coatings for effectively combating biomaterial-associated infections.

## 2. Materials and Methods

### 2.1. Materials and Reagents

Copper (II) chloride dihydrate (CuCl_2_·2H_2_O), polyvinylpyrrolidone (PVP, MW of ~10,000), hydrogen peroxide (H_2_O_2_, 30%), sodium hydroxide (NaOH), sulfuric acid (H_2_SO_4_), and potassium permanganate (KMnO_4_) were purchased from Sinopharm Group Co., Ltd., Shanghai, China. PCL powders (200 mesh, MW of ~80,000) were provided by Nature Works, Minneapolis, MN, USA.

### 2.2. Sample Preparation

CuO_2_ was synthesized according to Lin et al. [14] with slight modifications. To begin, 5 g PVP was added into 50 mL of aqueous solution containing 0.05 M CuCl_2_. After that, 50 mL of 0.10 M NaOH and 5 mL of 30% H_2_O_2_ were sequentially incorporated into the above mixture solution. After stirring for 30 min, the resulting nanoparticles were separated and purified to obtain the CuO_2_ powder after freeze-drying.

**Figure 1:**
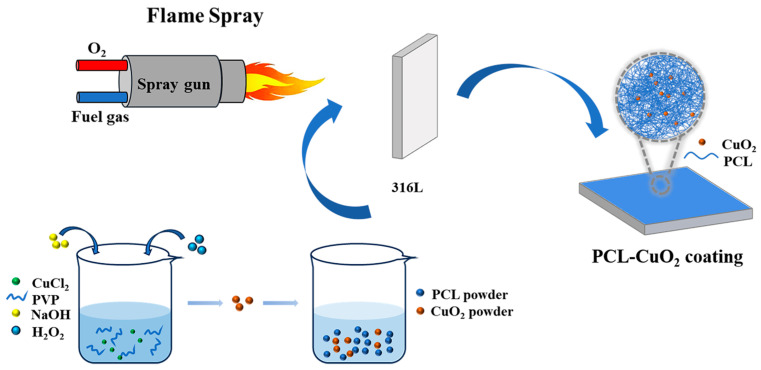
Schematic diagram of coatings prepared by liquid flame spraying.

PCL powders were suspended in 50:50 (*v*/*v*) ethanol/water, and then 0.1%, 0.3%, and 0.6% (*wt*/*wt*) CuO_2_ powders relative to PCL powders were added to prepare the PCL-CuO_2_ coatings. The coatings were prepared by flame spraying (CDS 8000, Castolin, Kriftel, Germany) on 316 L stainless-steel plates with dimensions of 25 × 20 × 2.0 mm [30]. The suspension was injected into the flame using a homemade spray atomizer. Acetylene was used as the fuel gas, with a flow rate of 1.5 Nm^3^/h and a working pressure of 0.1 MPa. Oxygen was used as the combustion gas, with a flow rate of 2.5 Nm^3^/h and a working pressure of 0.5 MPa. The spray distance was 200 mm. The thicknesses of PCL and PCL-CuO_2_ coatings were about 320–380 μm, measured by the coating thickness gauge. A schematic diagram of the preparation of the coating by liquid flame spraying is shown in Figure 1.

### 2.3. Sample Characterization

The microstructures of the powders and coatings were examined using a field emission scanning electron microscope (SEM, S4800, Hitachi, Tokyo, Japan). The cross sections of the coatings were characterized using an energy dispersive X-ray detector (EDX, XFlash 6–100, Bruker, Ettlingen, Germany). The particle size and zeta potential of CuO_2_ were measured by nanometer particle size analyzer (Litesizer 500, Anton Paar, Graz, Austria). Chemical composition was characterized using X-ray photoelectron spectroscopy (XPS, AXIS ULTRA DLD, Shimadzu, Kyoto, Japan). The X-ray diffraction (XRD) patterns were obtained on a D8-Advance X-ray diffractometer (Bruker, Germany), with Cu Kα radiation at a voltage of 40 kV and a tube current of 40 mA. Chemistry of the powders and coatings was detected by Fourier transform infrared spectroscopy (FT-IR, Nicolet iS50, Thermo Scientific, Waltham, MA, USA), operated at a spectral resolution of 4 cm^−1^ with a scan range of 4000~400 cm^−1^.

### 2.4. Colorimetric Determination of Peroxo Groups

KMnO_4_ solution is a strong purplish-red oxidizer that can oxidize H_2_O_2_, thus causing the purplish-red color to fade [14]. KMnO_4_ was dissolved in 0.1 M aqueous H_2_SO_4_ solution to obtain a concentration of 50 μg/mL, and then the acidic KMnO_4_ solution was treated with H_2_O (control), H_2_O_2_, Cu(OH)_2_, freshly synthesized CuO_2_, and the CuO_2_ suspension placed at room temperature for a short period, consecutively. After 10 min of incubation, photographs were taken to record the fading, and UV-vis spectra were examined at 400–650 nm.

### 2.5. pH-Responsive Release of Copper Ions

The areas surrounding the coating and the back of the substrate were sealed with epoxy resin, ensuring that only the coating surface remained exposed. For the release analysis, three parallel samples were used for each group of PCL-CuO_2_ composite coatings and each pH condition. Generally, coating samples were individually immersed in 15 mL PBS buffer at pH 7.4 or pH 5.5 in 50-mL falcon tubes with gentle shaking at 37 °C. At predetermined time intervals, 6 mL solution was collected with the addition of 6 mL fresh PBS. The content of copper ions was determined using inductively coupled plasma optical emission spectroscopy (ICP-OES; SPECTRO ARCOS, SPECTRO Analytical Instruments, Kleve, Germany).

### 2.6. In Vitro Antibacterial Effect of PCL-CuO_2_ Coatings

*Escherichia coli* (*E. coli*, ATCC25922) and *Staphylococcus aureus* (*S. aureus*, ATCC6538) were used to evaluate the antimicrobial effect of the coatings. We performed the tests according to the Japanese Industrial Standard (JIS) Z 2801 (ISO 22196:2011 [31], measurement of antibacterial activity on plastics and other nonporous surfaces) with some modifications [7]. Three parallel samples of each group of coatings were placed in sterile 6-well plates, and the bacteria cultured to logarithmic growth phase were diluted to about 1×10^6^ CFU/mL by gradient dilution in PBS at pH 7.4 or pH 5.5. Then, 10 μL bacterial suspension was dropped on the coating surface and covered with a sterile polyethylene film (5 × 5 mm) and then incubated at 37 °C with a relative humidity of at least 95% for 2 h. Afterward, bacteria were collected by washing with 1 mL PBS buffer and used for determination of survival rate by standard plate counting method. LB medium was used to grow *E. coli*, and TSB was used to grow *S. aureus*. The bactericidal effect can be calculated using the formula: R = (N_0_ − N_1_)/N_0_ × 100%, where N_0_ represents the number of bacterial colonies in the control group and N_1_ represents the number of bacterial colonies in the experimental group.

## 3. Results and Discussion

### 3.1. Morphological Characterization of PCL and CuO_2_ Powders

**Figure 2:**
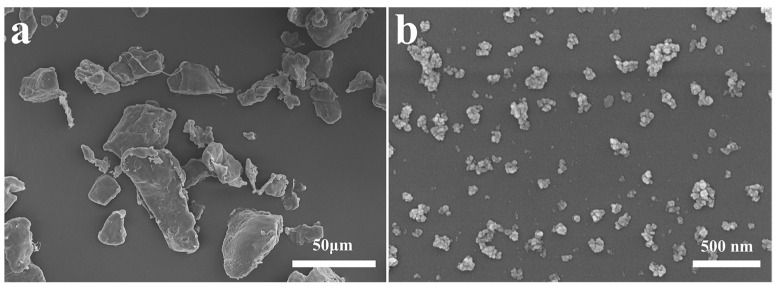
SEM images of (**a**) PCL powders and (**b**) CuO_2_ powders.

The morphology of commercially available PCL powders and synthesized nanosized CuO_2_ particles was examined by SEM analysis. The PCL powders showed irregular morphology and a wide size range, roughly from 10 to 60 μm (Figure 2a). The synthesized CuO_2_ particles were formed by aggregation of low nanosized particles and showed irregular shape and good dispersion (Figure 2b).

### 3.2. The Particle size and Zeta Potential of CuO_2_

**Figure 3:**
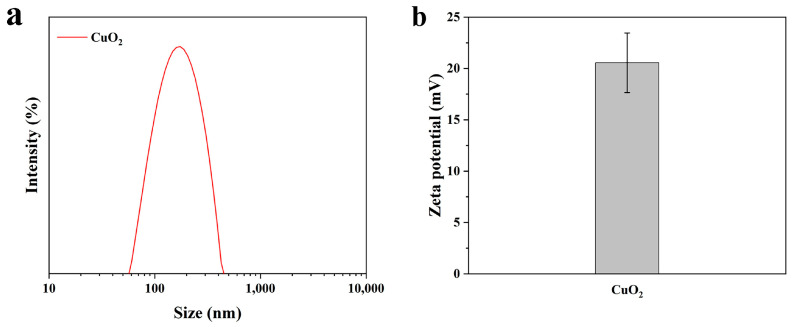
The particle size distribution (**a**) and zeta potential (**b**) of synthesized CuO_2_.

The particle size analysis showed that the average hydrodynamic diameter of the CuO_2_ was 163 ± 1.80 nm (Figure 3a). The results are consistent with the SEM results described above. Zeta potential is a good indicator of the magnitude of electrostatic interactions between dispersed particles and can be used as a reference for the stability of nanoparticle dispersions [32]. The average zeta potential carried by CuO_2_ was 20.6 ± 2.9 mV (Figure 3b), indicating that it has good dispersibility.

### 3.3. XPS Analysis of CuO_2_ Powders

**Figure 4:**
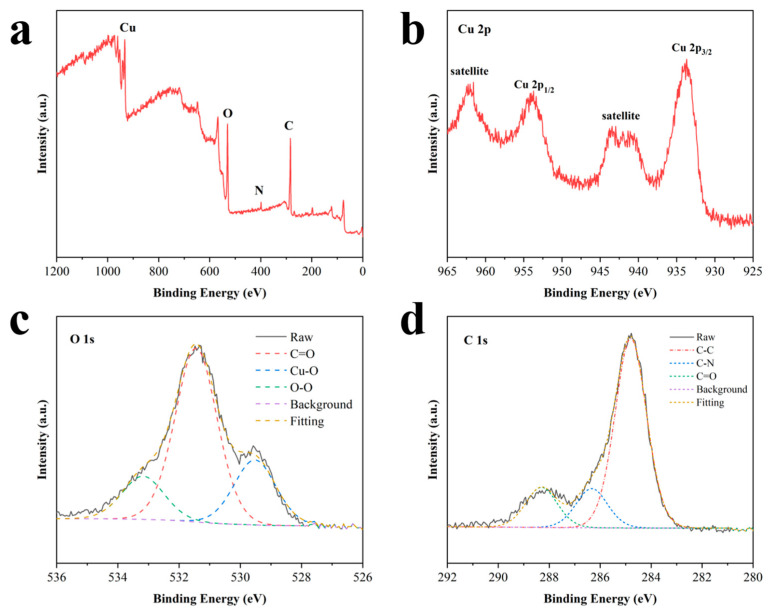
(**a**) XPS spectrum of synthesized CuO_2_ powders. (**b**–**d**) XPS expanded patterns of Cu 2p, O 1s, and C 1s.

The X-ray photoelectron (XPS) spectrum of the fully scanned region of CuO_2_ powders exhibited characteristic peaks of C 1s, N 1s, O 1s, and Cu 2p (Figure 4a). The peaks of C 1s and N 1s indicated the presence of PVP. The XPS spectrum of Cu 2p displayed characteristic peaks at 953.9 eV and 933.6 eV, respectively, accompanied by two satellite peaks at 962.1 eV and 942.1 eV, respectively, indicating that the valence state of copper in CuO_2_ is +2 [14] (Figure 4b). Furthermore, the O 1s XPS spectrum showed three distinct peaks at 529.5, 531.5, and 533.0 eV, ascribed to Cu-O, C=O, and O-O bonds, respectively [33]. The presence of peroxo groups in the synthesized CuO_2_ powder was confirmed by the presence of the O-O bond (Figure 4c). The XPS spectrum of C 1s showed three characteristic peaks at 284.8, 286.3, and 288.3 eV, which were assigned to C-C, C-N, and C=O, respectively [34] (Figure 4d). The above indicated the successful preparation of CuO_2_ nanoparticles.

### 3.4. Potassium Permanganate Colorimetric Analysis of Synthesized CuO_2_

**Figure 5:**
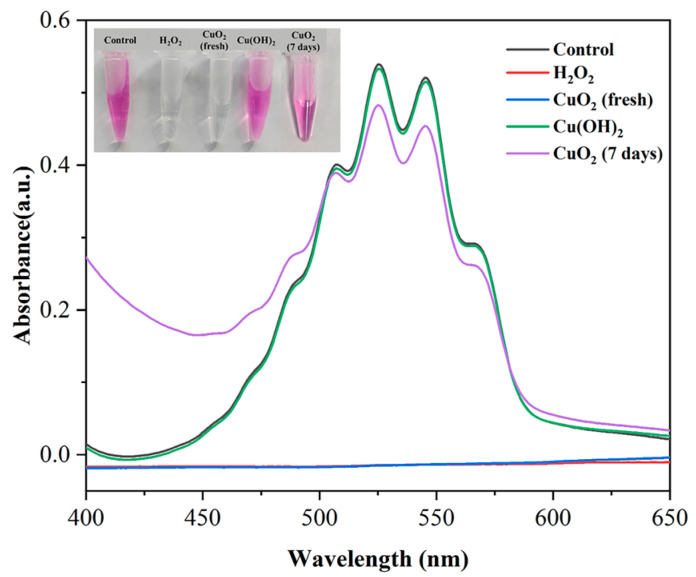
Colorimetric analysis demonstrating the presence of peroxo groups in CuO_2_ and its stability. CuO_2_ (fresh), freshly synthesized CuO_2_. CuO_2_ (7 days), CuO_2_ suspension left at room temperature for 7 days.

Furthermore, a KMnO_4_-based colorimetric method was used to examine the synthesized CuO_2_ powders. The absorption peaks of MnO_4_^−^ disappeared when mixed with H_2_O_2_ or the synthesized CuO_2_ powder but remained when mixed with H_2_O or Cu(OH)_2_ (Figure 5). It also suggests the presence of peroxo groups in the synthesized CuO_2_ powders, which is consistent with the above XPS results. However, CuO_2_ in water is unstable and easily decomposed [20]. As shown in Figure 5, the CuO_2_ suspension almost completely lost the ability to decolorize potassium permanganate after sitting at room temperature for 7 days, indicating the fast decomposition of CuO_2_.

### 3.5. SEM and EDX Analysis of the PCL and PCL-CuO_2_ Coatings

**Figure 6:**
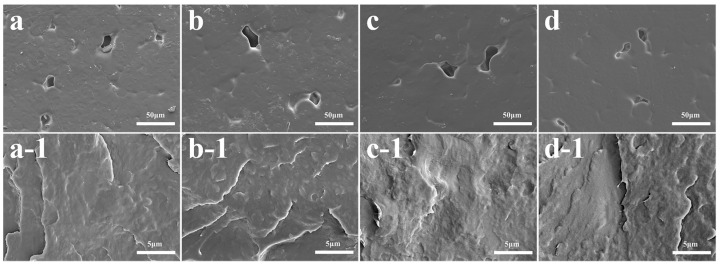
SEM images of (**a**) the PCL coating, (**b**) the PCL-0.1% CuO_2_ coating, (**c**) the PCL-0.3% CuO_2_ coating, and (**d**) the PCL-0.6% CuO_2_ coating. (**a-1**–**d-1**) The fracture surfaces morphology of the corresponding coatings.

**Figure 7:**
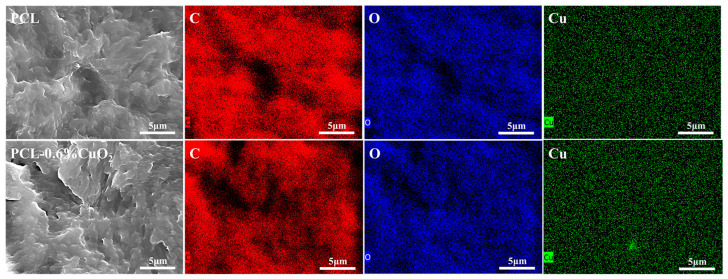
EDX results of the PCL coating and the PCL-0.6% CuO_2_ coating.

Figure 6a–d demonstrated that PCL and PCL-CuO_2_ coatings had smooth surfaces with visible pores. This might result from the fast evaporation of deionized water or ethanol during the manufacturing process [35]. There were no visible CuO_2_ particles on the surface or cross-section, which might be due to the homogeneous entrapment of PCL [36], as shown in Figure 6(a–d,a-1–d-1). These results demonstrated that the addition of CuO_2_ has no significant impact on either the surface or internal structure of the PCL coating. Furthermore, an enrichment of Cu was shown in the PCL-0.6% CuO_2_ coating in Figure 7, indicating the incorporation of CuO_2_ nanoparticles within the PCL matrix.

### 3.6. XRD Analysis of the Powders and Coatings

**Figure 8:**
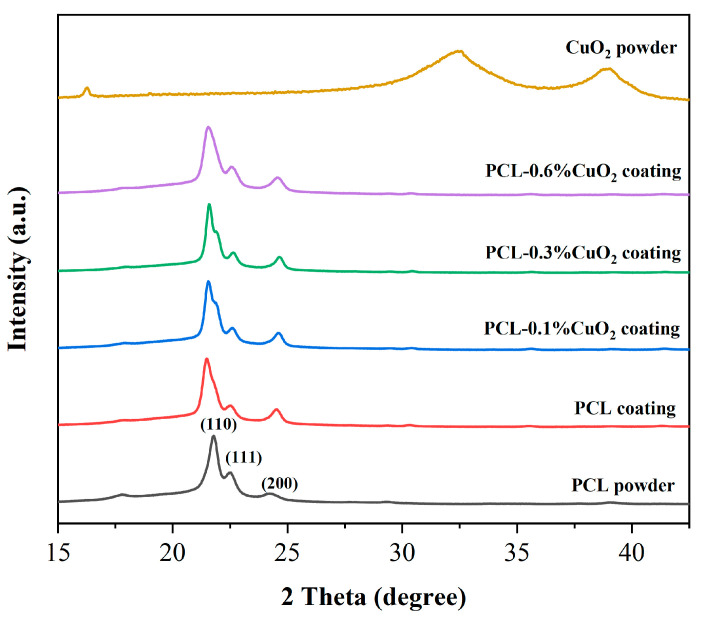
XRD patterns of PCL powders, synthesized CuO_2_ powders, the PCL coating, and PCL-CuO_2_ composite coatings.

In Figure 8, the XRD pattern analysis showed that the PCL powder exhibited intense diffraction peaks at 21.8°, 22.5°, and 24.2°, which are assigned to the planes (110), (111), and (200) of PCL, respectively [37]. The XRD pattern of the synthesized CuO_2_ nanoparticles was consistent with that reported in the literature, with two envelope peaks at 32.3° and 38.8° [33], indicating poor crystallization of the synthesized CuO_2_ [38]. These two envelope peaks were not observed in the PCL-CuO_2_ composite coating, which might be due to the low content of CuO_2_. These results indicated the successful synthesis of CuO_2_ nanoparticles and fabrication of PCL-CuO_2_ coatings.

### 3.7. FT-IR Analysis of the Powders and Coatings

**Figure 9:**
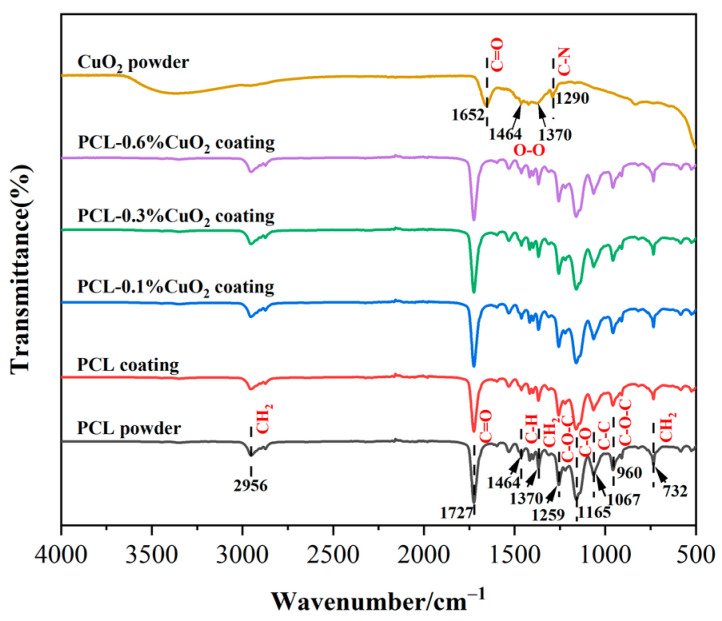
FT-IR spectra of PCL powders, CuO_2_ powders, the PCL coating, and PCL-CuO_2_ composite coatings.

The FT-IR spectra of PCL powder, CuO_2_ powder, PCL coating, and PCL-CuO_2_ composite coatings were analyzed, as shown in Figure 9. For PCL powder, the peak at 2956 cm^−1^ was the asymmetric stretching vibration of CH_2_, and the characteristic peak at 1727 cm^−1^ was the stretching vibration of the C=O group. The peak of stretching vibration of C-H at 1464 cm^−1^, 1370 cm^−1^ belongs to CH_2_, and the peak of asymmetric stretching vibration of C-O-C group at 1259 cm^−1^, 1165 cm^−1^ belongs to C-O. The peaks at 1067 cm^−1^, 960 cm^−1^, and 732 cm^−1^ represented the C-C, C-O-C, and CH_2_ vibration peaks, respectively [39]. There was no significant difference in the position of the infrared absorption peak between the PCL powder and the prepared coating, indicating that the chemical composition of the PCL coatings prepared by suspension flame spraying was not changed. The characteristic peaks at 1652 cm^−1^ and 1290 cm^−1^ in the CuO_2_ powder are attributed to the tensile vibration between C=O and C-N in PVP [40], and the two small peaks displayed at 1464 cm^−1^ and 1370 cm^−1^ are the characteristic peaks of peroxide group (O-O) [41]. The above characteristic peaks of the CuO_2_ powder were not detected in the PCL-CuO_2_ coatings, which might be due to the low content of CuO_2_ in the composite coatings.

### 3.8. pH-Responsive Release of PCL-CuO_2_ Coatings

**Figure 10:**
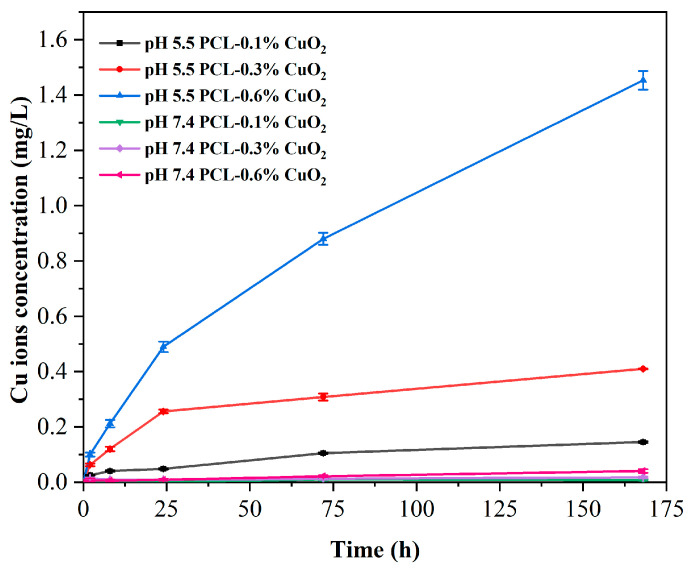
Cumulative release of Cu^2+^ from PCL-CuO_2_ coatings.

Under weakly acidic conditions, CuO_2_ decomposes to produce Cu^2+^ and H_2_O_2_, which further produces reactive oxygen species via the Fenton reaction [14]. The release of Cu^2+^ from the PCL-CuO_2_ coatings was examined under different pH conditions (Figure 10). As expected, increasing Cu^2+^ release with the content of CuO_2_ was clearly observed at pH 5.5, while the release at pH 7.4 was negligible. At pH 5.5, the copper ion release from PCL-0.1% CuO_2_ coating for 7 days was 0.15 mg/L; from PCL-0.3% CuO_2_ coating, it was 0.41 mg/L; and from PCL-0.6% CuO_2_ coating, it was 1.45 mg/L. However, the release of copper ions from PCL-0.1% CuO_2_, PCL-0.3% CuO_2_, and PCL-0.6% CuO_2_ coatings at pH 7.4 for 7 days were only 0.01, 0.02, and 0.04 mg/L, respectively. The above indicated that the PCL-CuO_2_ coatings showed sustained release in an acid-responsive and dose-dependent manner. A previous study reported that a concentration of Cu^2+^ of no more than 9 ppm showed no significant effect on the growth of cells compared with normal conditions [42]. In this study, the release of Cu^2+^ was very slow, and the concentration of Cu^2+^ after 7 days continuous release was significantly lower than 9 ppm, suggesting high biocompatibility of the PCL-CuO_2_ coatings.

**Figure 11:**
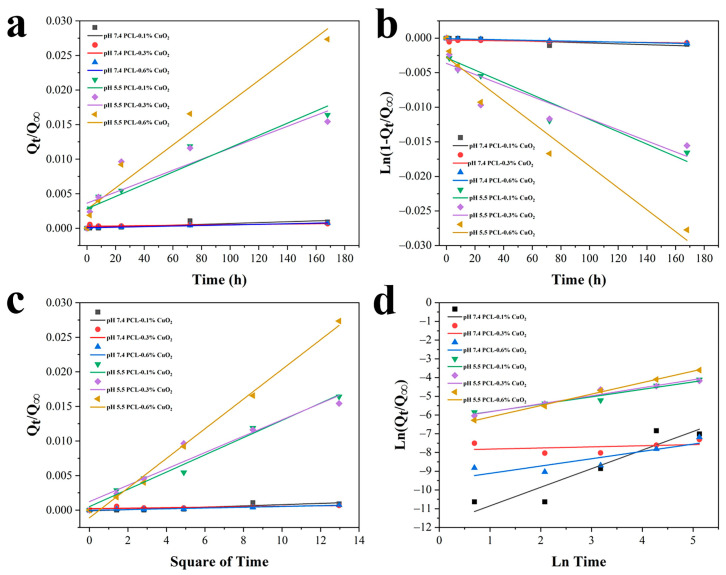
Characteristics of the Cu^2+^ release behaviors of the PCL-CuO_2_ coatings by applying the zero-order model (**a**), the first-order model (**b**), the Higuchi model (**c**), and the Korsmeyer–Peppas model (**d**).

Furthermore, the data were fitted with four commonly used drug release models (Figure 11a–d) and listed in Table 1. It was noted that, under pH 5.5, the release for the PCL-0.3% CuO_2_ and PCL-0.6% CuO_2_ coatings fitted the Korsmeyer–Peppas model, with the highest linearity correlation coefficient (R^2^ = 0.964, 0.997). The release exponent n for PCL-0.3% CuO_2_ was ≤0.45, indicating the drug release mechanism follows Fick’s laws of diffusion. On the contrary, the release exponent n for PCL-0.6% CuO_2_ was higher than 0.45, suggesting a combined erosion and diffusion release mechanism, named non-Fickian transport. The release of the PCL-0.1% CuO_2_ coating was best interpreted by the Higuchi equation (R^2^ = 0.984), indicating a relatively slower diffusion from the PCL matrix. However, it was difficult to define the release models of the PCL-CuO_2_ coatings under pH 7.4, which generally showed an R^2^ value lower than those under pH 5.5. This might result from the extremely slower release of the composite coatings under pH 7.4. The complexity of the release mechanisms for these coatings might be due to the combined impact of CuO_2_ diffusion from the coating and the decomposition reaction of CuO_2_.

### 3.9. In Vitro Antibacterial Properties of PCL-CuO_2_ Coatings

**Figure 12:**
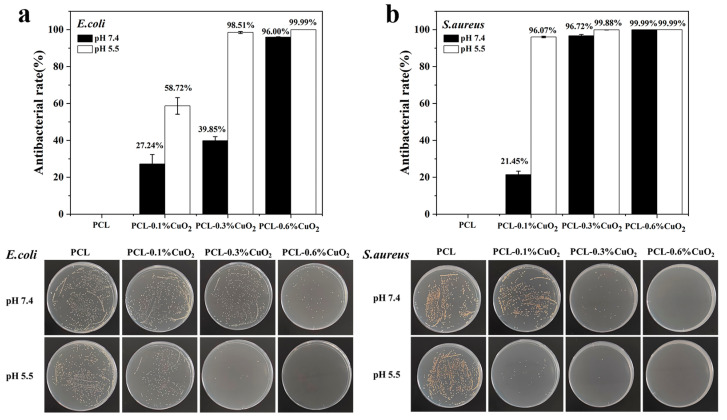
In vitro antibacterial activity test of PCL-CuO_2_ coatings at pH 7.4 and pH 5.5. Antimicrobial effect of (**a**) *E. coli* and (**b**) *S. aureus* after 2 h incubation on the PCL-CuO_2_ composite coatings.

The decomposition of CuO_2_ displays a pH-responsive manner and can continuously release copper ions and H_2_O_2_ under weakly acidic conditions. Therefore, the antibacterial effect of the PCL-CuO_2_ composite coating was examined at pH 5.5 and 7.4 using *E. coli* and *S. aureus*. It was found that the composite coatings exhibited a significantly higher antibacterial effect against both *E. coli* and *S. aureus* at pH 5.5 compared to pH 7.4 (Figure 12a,b). Furthermore, the antibacterial effect of the composite coatings was dose-dependent and reached over 99.99% killing efficacy against both *E. coli* and *S. aureus* for 0.6% CuO_2_ content at pH 5.5. Moreover, the composite coatings displayed superior killing efficacy for *S. aureus* compared to *E. coli* under both pH conditions, which might be due to there being a difference in the antimicrobial activity of metal nanoparticles depending on the bacterial wall structure [7]. Gram-positive bacteria with higher peptidoglycan and cell wall protein content are more sensitive to copper [16,43]. Excitingly, there is no significant change in the antimicrobial effect of these coatings after 10 months (Figure S1), suggesting the excellent stability of CuO_2_ within the PCL-CuO_2_ coatings.

## 4. Conclusions

In this research, PCL-CuO_2_ composite coatings with pH-responsive antimicrobial properties were fabricated using a suspension flame spraying technique. Morphology and chemical characterization confirmed the well-maintained PCL matrix and the successful incorporation of CuO_2_ nanoparticles in the composite coatings. The release study found that the PCL-CuO_2_ coatings showed sustained release in an acid-responsive and dose-dependent manner. The composite coating with 0.6% (*w*/*w*) CuO_2_ exhibited over 99.99% antibacterial effect against *E. coli* and *S. aureus* under a mildly acidic (pH 5.5) condition. The CuO_2_ nanoparticles in the PCL-CuO_2_ composite coatings showed significantly enhanced stability in comparison with fast decomposition in an aqueous solution and still exhibited potent antimicrobial efficacy after 10 months storage. Our cost-effective fabrication method of pH-responsive antimicrobial coatings provides a new solution for the development of biomedical materials for various applications.

## Figures and Tables

**Table 1:** The variables calculated from the release kinetics of Cu^2+.^

Sample		7.4 0.1%	7.4 0.3%	7.4 0.6%	5.5 0.1%	5.5 0.3%	5.5 0.6%
Zero-order model	K_0_ (×10^−5^)	0.62	0.24	0.42	8.84	7.94	15.55
	R^2^	0.691	0.449	0.971	0.902	0.774	0.953
First-order model	K_1_ (×10^−5^)	−0.62	−0.24	−0.42	−8.92	−8.01	−15.78
	R^2^	0.691	0.449	0.971	0.904	0.776	0.955
Higuchi model	K_HI_ (×10^−5^)	0.90	0.35	0.55	12.5	11.8	21.5
	R^2^	0.786	0.521	0.944	0.984	0.943	0.995
Korsmeyer–Peppas model	K_KP_ (×10^−5^)	0.08	3.71	0.75	20.29	18.36	12.31
	R^2^	0.879	0.108	0.764	0.957	0.964	0.997
	n	0.99	0.06	0.39	0.40	0.43	0.61

## Data Availability

There is no data used in the study.

## Supplementary Materials

The following supporting information can be downloaded at: https://www.mdpi.com/article/10.3390/ma17112666/s1, Figure S1. In vitro antibacterial activity test of PCL-CuO_2_ coatings at pH 7.4 and pH 5.5 after 10 months of storage at room temperature. Antimicrobial effect of (a) *E. coli* and (b) *S. aureus* after 2 h incubation on the PCL-CuO_2_ composite coatings.
