# Supplementary material for: A pH-Responsive Polycaprolactone–Copper Peroxide Composite Coating Fabricated via Suspension Flame Spraying for Antimicrobial Applications
Source: Materials (Basel). 2024 Jun 1;17(11):2666. doi: 10.3390/ma17112666 (PMC11173732; doi:10.3390/ma17112666)
Supplement: Supplementary file 1 [file materials-17-02666-s001.zip › materials-3003999-supplementary.pdf]

materials

Supplementary Materials

A pH-Responsive Polycaprolactone–Copper Peroxide Composite Coating Fabricated via Suspension Flame Spraying for Antimicrobial Applications

Tingting Cui [1,2], Daofeng Zhou [1,2], Yu Zhang [1,2], Decong Kong [1,2], Zhijuan Wang [1,2], Zhuoyue Han [1,2], Meiqi Song [1,2], Xierzhati Aimaier [1,2], Yanxin Dan [3], Botao Zhang [1,2,4,*] and Hua Li [1,2,4,*]

[1] Cixi Biomedical Research Institute, Wenzhou Medical University, Wenzhou 325035, China
[2] Institute of Biomedical Engineering, Ningbo Institute of Materials Technology and Engineering, Chinese Academy of Sciences, Ningbo 315201, China
[3] Graduate School of Engineering, Tohoku University, Sendai 980-8577, Japan
[4] Zhejiang-Japan Joint Laboratory for Antibacterial and Antifouling Technology, Ningbo Cixi Institute of Biomedical Engineering, Ningbo 315201, China
* Correspondence: zhangbotao@nimte.ac.cn (B.Z.); huali@nimte.ac.cn (H.L.)

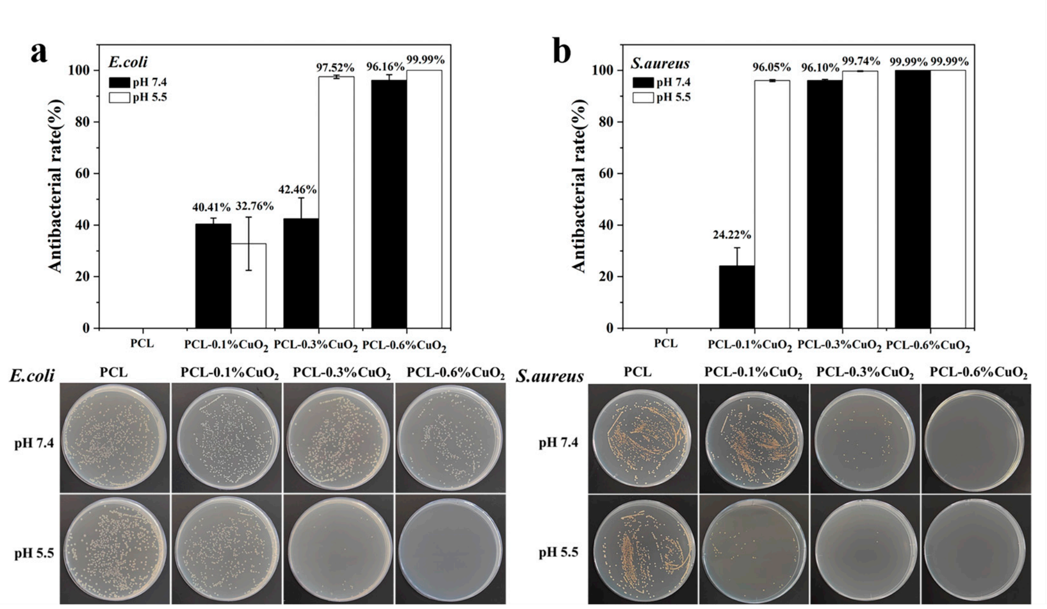

Figure S1. In vitro antibacterial activity test of PCL-CuO_2 coatings at pH 7.4 and pH 5.5 after 10 months of storage at room temperature. Antimicrobial effect of (a) *E. coli* and (b) *S. aureus* after 2 hours incubation on the PCL-CuO_2 composite coatings.
